# The Spatial and Temporal Transcriptomic Landscapes of Ginseng, *Panax ginseng* C. A. Meyer

**DOI:** 10.1038/srep18283

**Published:** 2015-12-11

**Authors:** Kangyu Wang, Shicui Jiang, Chunyu Sun, Yanping Lin, Rui Yin, Yi Wang, Meiping Zhang

**Affiliations:** 1 College of Life Science, Jilin Agricultural University, Changchun 130118, Jilin, China

## Abstract

Ginseng, including Asian ginseng (*Panax ginseng* C. A. Meyer) and American ginseng (*P. quinquefolius* L.), is one of the most important medicinal herbs in Asia and North America, but significantly understudied. This study sequenced and characterized the transcriptomes and expression profiles of genes expressed in 14 tissues and four different aged roots of Asian ginseng. A total of 265.2 million 100-bp clean reads were generated using the high-throughput sequencing platform HiSeq 2000, representing >8.3x of the 3.2-Gb ginseng genome. From the sequences, 248,993 unigenes were assembled for whole plant, 61,912–113,456 unigenes for each tissue and 54,444–65,412 unigenes for different year-old roots. We comprehensively analyzed the unigene sets and gene expression profiles. We found that the number of genes allocated to each functional category is stable across tissues or developmental stages, while the expression profiles of different genes of a gene family or involved in ginsenoside biosynthesis dramatically diversified spatially and temporally. These results provide an overall insight into the spatial and temporal transcriptome dynamics and landscapes of Asian ginseng, and comprehensive resources for advanced research and breeding of ginseng and related species.

Medicinal plants are important to human health and medicine, but most of them are significantly understudied, especially in modern genetics and genomics. Ginseng, including American ginseng (*Panax quinquefolius* L.) and Asian ginseng (*P. ginseng* C.A. Meyer), is the number one of the medicinal herbs in North America and Asia^1^. Asian ginseng grown in northeast China, often known as Jilin ginseng, accounts for 65% of the world production, thus becoming a model species for medicinal chemistry, genetics and genomics research of ginseng and many other related medicinal plants. Ginseng has been historically widely used in human health and medicine, especially in Asia^2^. It has been documented that ginseng has several human health and medical values, including, but not limited to, providing energy boost, lowering blood sugar and cholesterol levels, reducing stress, promoting relaxation, treating diabetes, and treating man’s sexual dysfunction^3^,^4^,^5^,^6^. The ginsenosides of ginseng have been shown to be its major medically bioactive compounds. They are triterpenoid saponins found nearly exclusively in ginseng; therefore, they have been a major focus of ginseng research^7^. Triterpenoids are synthesized through one of major isoprenoid biosynthetic pathways^8^. Triterpenes are one of the most important classes of natural products, glycosides saponins of which have been important natural medicines^9^. To date, more than 150 natural ginsenosides have been isolated from *Panax* species and most of them can be classified into two groups, based on the skeleton of their aglycones, namely dammarane type and oleanane type^10^,^11^,^12^. The dammarane type consists mainly of three varieties according to their genuine aglycone moieties: 20S-protopanaxadiol (PPD), 20S-protopanaxatriol (PPT) and ocotillol^13^,^14^,^15^.

Genome research has been shown in many plants and animals to be significant for enhanced isolation, characterization and utilization of genes of economic importance. Nevertheless, because ginseng is a perennial allotetraploid, containing 2*n* = 48 chromosomes with a genome size of about 3.2 Gb^16^, it is a great challenge for ginseng genomics research, especially at the genome level. Ginseng genomics research has been restricted to development of a limited number of DNA markers^17^,^18^,^19^,^20^ and ESTs^21^,^22^, construction of BAC^16^ and BIBAC^23^ libraries, and transcriptome sequencing of limited tissues, especially roots^24^,^25^,^26^,^27^. The shortage of comprehensive genomic knowledge, resources and tools is significantly limiting its advanced research, modernized breeding and application of economical genes.

This study, using the next-generation high-throughput sequencing technologies^28^, sequenced and characterized the transcriptomes and the expression profiles of genes expressed in 14 different tissues and four different year-old roots sampled from a widely growing Jilin ginseng cultivar, Damaya. From the gene sequence reads and expression data, we assembled comprehensive unigene sets for Asian ginseng, functionally categorized them *in silico*, and characterized their expression profiles spatially and temporally and co-expression networks. We also identified the genes involved in triterpenoid biosynthesis that is believed playing an important role in ginsenoside biosynthesis, and analyzed their activities and co-expression networks in different tissues and different year-old roots. Therefore, the results and findings of this study provide a first overall overview of ginseng gene functions and expression activities in different tissues and at different developmental stages, and resources essential for advanced research and applications of economic genes of ginseng and related species.

## Results

### Development and annotation of comprehensive unigene sets for Asian ginseng: whole plant, individual tissues and different year-old roots

The medical values of ginseng increase rapidly as its age increases, but ginseng for commercial production is usually harvested when it is 4–5 years old. Therefore, four-year-old plants of Jilin ginseng cv. Damaya were sampled at the fruit ripening stage. Since different parts, including those from same organ such as root system, are significantly different in the content of ginsenosides (data not shown), we divided the plant into 14 parts (hereafter named as tissues) for transcriptome analysis: fiber root (a), leg root (b), main root epiderm (c), main root cortex (d), rhizome (e), arm root (f), stem (g), leaf peduncle (h), leaflet pedicel (i), leaf blade (j), fruit peduncle (k), fruit pedicel (l), fruit flesh (m) and seed (n) (Figure S1). The 14 tissues together were used to represent whole plant (hereafter named as whole plant). Moreover, since root systems are major medical products of ginseng and the medical values of ginseng increase rapidly as their ages increase, they were also collected from 5-, 12-, 18- and 25-year old plants. The transcriptomes of all tissues and roots were subjected to sequencing and digital gene expression profiling. High-quality 100-bp clean reads from 10.7 to 16.8 million, with an average of 14.3 million, were obtained for each of the 14 tissues. This coverage of RNA sequencing was proved to be sufficient for accurate gene expression profiling in our previous study (see Methods) and supported by another widely used digital gene expression profiling method – the serial analysis of gene expression (SAGE)^29^,^30^,^31^. In comparison, the coverage of the 14.3 million 100-bp clean reads was more than 10-fold higher than those widely used for SAGE^29^,^30^,^31^, if 70,000 unigene isoforms were assembled from each tissue or each developmental stage of roots and the transcripts of ginseng have an average full length of 1,500–2,500 bp as those of maize and cotton^32^,^33^.

A total of 199.0 million 100-bp clean reads were obtained from the 14 tissues, representing 19.9 Gb sequences that are equivalent to >6.2x of the ginseng 3.2-Gb genome. *De novo* assembly of the 199.0 million 100-bp clean reads resulted in a set of 248,993 unigenes corresponding to 248,993 isoforms or transcripts resulted from RNA alternative splicing, with an N50 of 1,572 bp and an average length of 936 bp (Table 1). The number of the transcripts was in consistence with the 232,702 transcript unigenes of Jayakodi *et al.*^27^ obtained from root. To further assess the assembly, we blasted a selection of approximately 1,000 random unigenes against the nucleotide sequences in GenBank. The results showed that over 98% of the unigenes hit to single loci of the sequences at an E-value ≤ 1.0E-05, suggesting that the unigene assembly is acceptable. These unigene isoforms were shown to correspond to 130,557 gene models, ranging from 1–136 isoforms per gene model with an average of 1.91 isoforms per gene model and 16.05% of the gene models having two or more isoforms (Figure S2). The assembly of the clean reads derived from each tissue resulted in 60,912–113,456 unigenes, with an N50 of 1,060–1,372 bp and an average length of 716–852 bp (Table 1). The assembly of the clean reads derived from each year-old root yielded 54,444–65,412 unigenes, with an N50 ranging from 1,157–1,352 bp and an average length from 668–823 bp (Table 1).

The unigene set derived from whole plant was then subjected to annotation against the NCBI Nr database. Because the unigene set might contain those derived from rRNA genes and long non-coding (lnc) RNAs, we blasted the ginseng unigene set against the rRNA database of all plant species available in GenBank and conducted ORF analysis. The results showed that 434 of the unigenes were derived from rRNA genes and 32,299 were from lncRNA genes. Therefore, 216,260 of the unigenes were likely derived from coding genes. Annotation showed that of the 216,260 unigenes, 170,088 (78.63%) were found to have significant similarity with protein sequences in the database at a cutoff of E-value ≤ 1e-05. This result is in agreement with the 94% of the Asian ginseng root transcriptome annotation rate^27^ because our unigene set of Asian ginseng was derived from 14 different tissues, most of which were analyzed for the first time in transcriptome. These unigenes were annotated with the genes of 29 species, of which over a half were annotated with the genes of *Vitis vinifera* (27%), *Solanum lycopersicum* (9%), *Aegilops tauschii* (9%) and *Theobroma cacao* (9%) in GenBank.

### *In silico* functional analysis of the unigene sets derived from whole plant, individual tissues and different year-old roots

**Figure 1:**
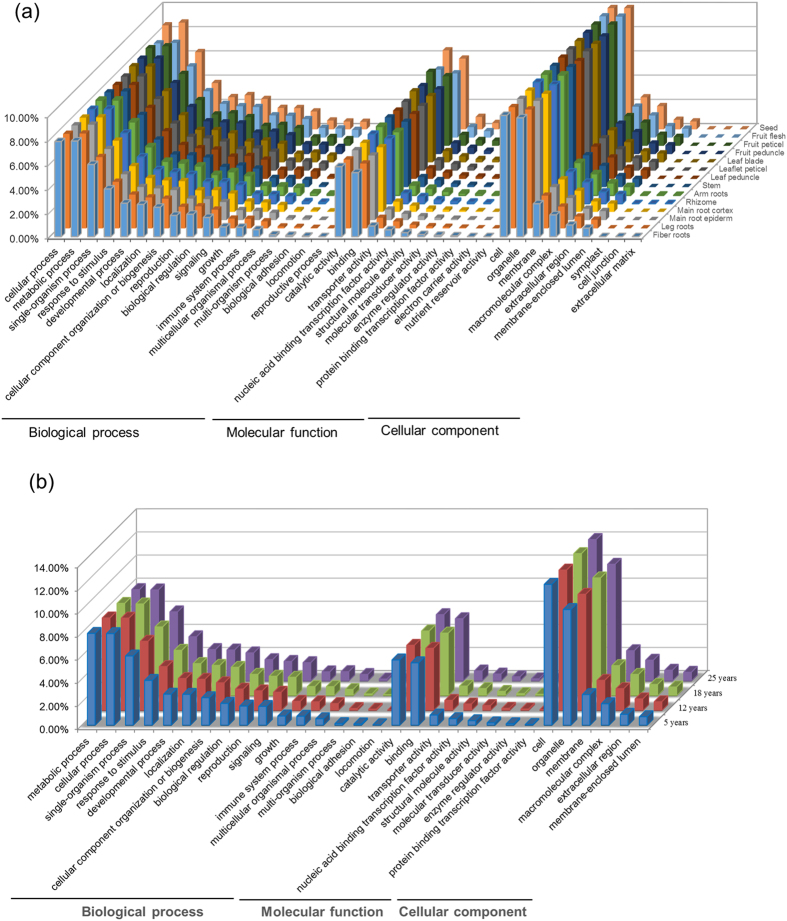
GO term categorization and comparison of genes expressed in (a) whole plant or different tissues and (b) different year-old roots. The number of gene GO terms categorized into each functional subcategories is present in the percentage of GO terms for that subcategory out of the total GO terms that the unigenes assigned to for whole plant (14 tissues) or each tissue, or different year-old roots (*z*-axis).

To have a first glimpse into the dynamics of the Asian ginseng transcriptome in a view of systems biology, we first categorized the unigene sets of whole plant, 14 tissues and four year-old roots, separately, by ontological (GO) analysis and KEGG pathway mapping. GO analysis assigned 109,781 of the annotated unigenes of whole plant to 716,067 GO terms, indicating that a number of the unigenes have two or more GO terms, under the functional categories of molecular function, cellular component and biological process that are further grouped into subcategories (Table 2, Fig. 1a). Of the unigenes of each tissue, 31,311–58,557 were each assigned to one or more GO terms, with a total of 182,789–385,996 GO terms for each tissue (Table 2, Fig. 1a). Of the unigenes of different year-old roots, 32,306–38,202 were each assigned to one or more GO terms, with a total of 217,553–254,454 GO terms for each aged root (Table 2, Fig. 1b). In comparison, the unigene sets have a very similar GO term profile of functional categorization, regardless of whether they were derived from whole plant, different tissues or different year-old roots (Fig. 1), suggesting that it is essential for maintaining the normal activities of a cell to have a consistent percentage of genes allocated to each functional category among different tissues and different developmental stages.

KEGG pathway mapping assigned 29,321 (26.7%) of the 109,781 unigenes of whole plant having GO term assignments to 35,819 enzymes (ECs) involved in 138 metabolic pathways. Of the pathways, ko00900 (terpenoid backbone biosynthesis) and ko00909 (sesquiterpenoid and triterpenoid biosynthesis) are likely to significantly contribute to ginsenoside biosynthesis^7^. KEGG pathway mapping assigned 8,851 (26.0%)–16,106 (27.5%) of the unigenes of each tissue having GO term assignments to 9,417–19,502 ECs involved in 133–138 metabolic pathways, and 8,409 (26.0%)–9,723 (25.5%) of the unigenes of each year-old root having GO term assignments to 10,238–11,773 ECs involved in 135–137 pathways. The number of pathways in which the unigenes of each tissue or each year-old root that had GO term assignments were involved was similar to that of whole plant (Table 2). These results support the above observation that it is essential for maintaining the normal activities of a cell to have a consistent percentage of genes involved in metabolic pathways among different tissues and different developmental stages.

**Figure 2:**
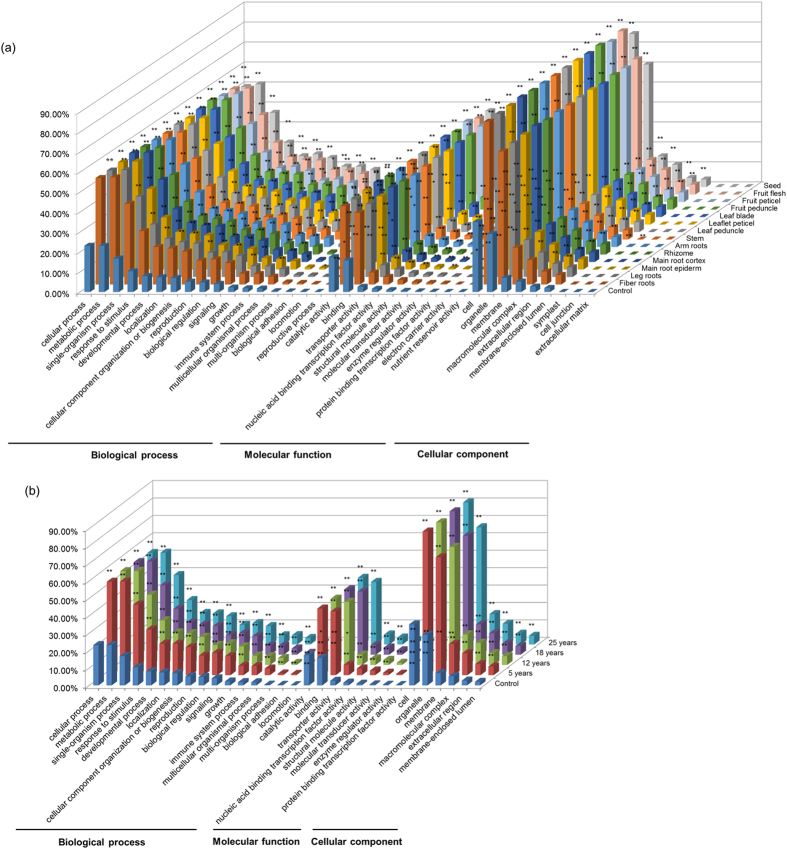
Enrichment of gene GO terms in (a) different tissues and (b) different year-old roots relative to control (whole plant). The number of gene GO terms categorized into each functional subcategories is present in the percentage of GO terms for that subcategory out of the total GO terms that the unigenes assigned to for whole plant (14 tissues) or each tissue, or different year-old roots (*z*-axis). The enrichments of the unigenes categorized into each subcategory of different tissues or different year-old roots were estimated by χ^2^ test using the percentage of the unigenes in the subcategory in the whole plant as the expected. *, a significance level of *P* ≤ 0.05 (FDR ≤ 0.063 for different tissues and FDR ≤ 0.053 for different year old roots); **, a significance level of *P* ≤ 0.01 (FDR ≤ 0.013 for different tissues and FDR ≤ 0.011 for different year old roots).

Furthermore, the numbers of unigenes allocated to different functional subcategories in a cell in different tissues and at different developmental stages of a same tissue (root) were tested with multiple testing correction against those of whole plant as controls. The results showed that the numbers of unigenes allocated to most of the 36 functional subcategories (GO term level 2) in different tissues, varying from 23 for leaf peduncle to 33 for arm root, were enriched (FDR ≤ 0.063). However, interestingly, every tissue had its own unique enrichment pattern distinguishing from those of other tissues (Fig. 2a). For different developmental stages of roots, the numbers of unigenes allocated to 27, 29, all and 28 of the 30 functional subcategories were enriched (FDR ≤ 0.053) for 5-, 12-, 18-, and 25-year old roots, respectively (Fig. 2b). The enrichment pattern was also unique for each developmental stage of roots.

### Expression profiles of the unigenes in different tissues and different year-old roots

**Figure 3:**
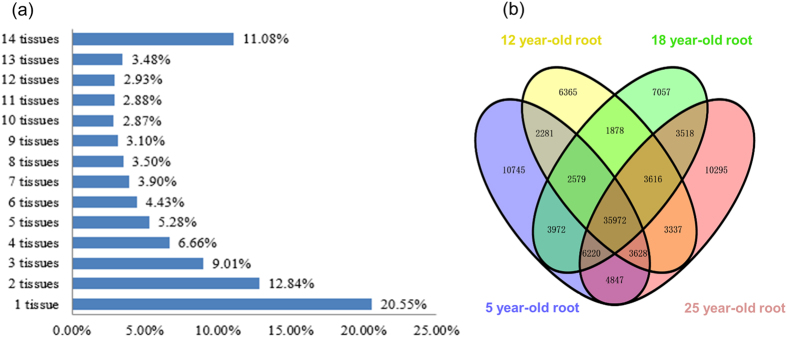
Percentage or number of unigenes specifically expressed in (a) 14 different tissues and (b) different year-old roots.

To further have an overview of their functional activities and expression action relationships, the expressions of the 248,993 unigenes in different tissues and different year-old roots were digitally measured and their expression relationships were characterized. If a gene was expressed only one or more of the tissues analyzed, we defined the gene as one or more tissues specifically expressed. The expressions of most unigenes varied by several fold among different tissues or through different stages of root growth and development (Table S1). For the 14 different tissues, a total of 51,169 unigenes were shown to be specifically expressed in one of them, representing 20.55% of the 248,993 unigenes (Fig. 3a). Of the one tissue-specifically expressed 51,169 unigenes, the numbers of unigenes varied by more than 37 fold, from 890 for rhizome to 33,169 for fruit peduncle. Of the 248,993 unigenes, only 27,596 (11.08%) expressed in all 14 tissues studied, indicating that most of the genes in ginseng only expressed in one or few of tissues (Table S2). A total of 151,602 unigenes were found to be expressed in 2–13 of the 14 tissues studied, together accounting for 60.89% of the 248,993 unigenes. The numbers of the unigenes specifically expressed in one tissue were much larger than those specifically expressed in two tissues. It was also observed that the number of unigenes specifically expressed in fruit pedicel was much larger than that specifically expressed in other tissues studied. Since the tissues were collected at the fruit ripening stage, the exceptionally larger number of unigenes specifically expressed in fruit pedicel may indicate that it is essential for rapid transportation of large amounts of nutrition to fruits from other tissues through fruit pedicels. For the four different year-old roots, from 6,365–10,745 unigenes were observed to specifically express at one of the four stages, with a descending order of 5-, 25-, 18- and 12-year-old roots. It appeared that the number of developmental stage-specific expressed unigenes varied during the process of root growth and development as a normal distribution, with the 12-year-old root having the most developmental stage-specific expressed genes. Of the unigenes, 35,972 (59.8%) were shared among the four developmental stages of roots (Fig. 3b). It was apparent that the percentage of unigenes commonly expressed at different stages of roots is much larger than that commonly expressed in different tissues.

**Figure 4:**
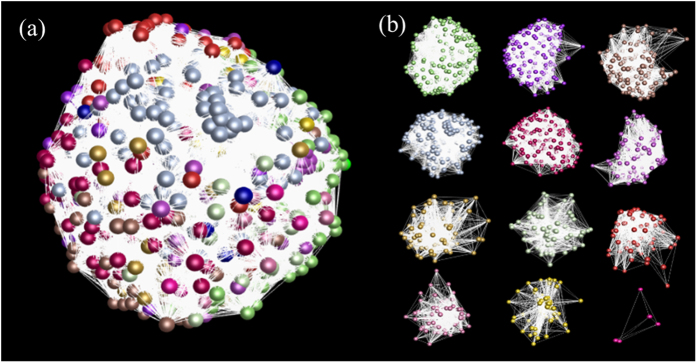
Example of co-expression network of unigenes. (**a**) Gene co-expression network constructed from 5,000 unigenes randomly selected from the 248,993 unigenes at a cutoff of *P* ≤ 0.05. Nodes = 5,000, edges = 971,280, clusters = 22. Different colors indicate different clusters that have closer interactions. The result indicates that the genes contained in a cell are somehow interacted in expression. (**b**) Example of different clusters of the unigenes grouped from the network shown in figure (**a**).

The expression relationships of the unigenes were determined by calculating correlation coefficients of gene co-expression, which are shown by gene co-expression networks. Since it was difficult to calculate the pairwise correlation coefficients of 248,993 unigenes [(248,993 × 248,993/2 = 3.1 × 10^10^ correlation coefficients)] using a current computing tool, we estimated the expression relationships of the genes in a cell by analyzing a random sample of 5,000 unigenes selected from the 248,993 unigene set. The co-expression network of the 5,000 unigenes is shown in Fig. 4. While a cutoff of *P* ≤ 0.05 was used for construction of the network, the number of edges (971,280) was 1.6 fold of the probability of gene co-expression by chance (*P* = 0.0000). Furthermore, we also constructed the network at a cutoff of *P* ≤ 0.001. A network consisting of 4,983 nodes, 60,049 edges and 1,508 clusters resulted. The number of edges (60,049) was 4.8 fold of the probability of gene co-expression by chance (*P* = 0.0000). These results indicate that all of the genes in a cell are somehow correlated in expression and those functionally more related have closer correlations in action to form clusters (shown as clusters in Fig. 4).

### Isolation and characterization of the genes likely involved in ginsenoside biosynthesis

**Figure 5:**
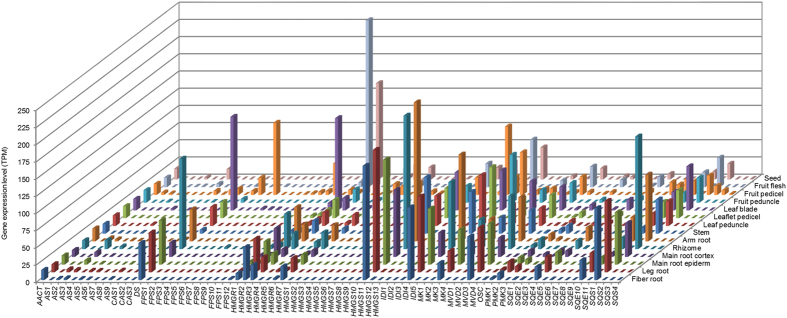
Expression variation of 78 genes likely involved in ginsenoside biosynthesis among 14 different tissues. *X-*axis indicates the candidate genes (see Tables S3, S4); *y*-axis on right indicates the 14 different tissues and *z*-axis is for the expression levels of each gene in different tissues.

**Figure 6:**
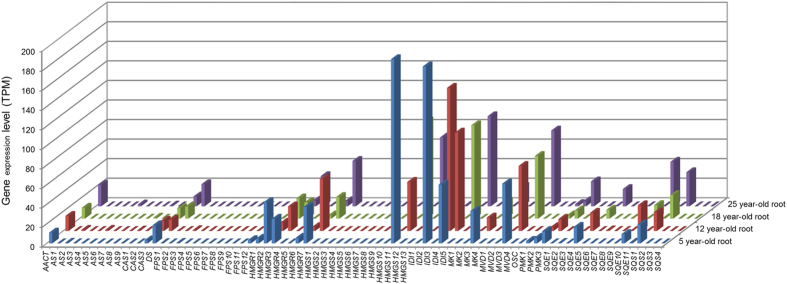
Expression variation of 78 genes likely involved in ginsenoside biosynthesis through different stages of root growth and development. *X-*axis indicates the candidate genes (see Tables S3, S4); *y*-axis on right indicates the four different year-old roots and *z*-axis is for the expression levels of each gene in different year-old roots.

As terpenoid backbone biosynthesis (ko00900), and sesquiterpenoid and triterpenoid biosynthesis (ko00909) likely significantly contribute to the biosynthesis of ginsenosides, the major medically bioactive compounds of ginseng^7^, we inventoried the unigenes mapped to the two pathways (see above). A total of 155 unigene isoforms were isolated and these unigene isoforms were shown to be derived from 78 gene models (Tables S3, S4). These 78 genes were found to encode 13 enzymes, with each enzyme encoded by one gene family consisting of 1–13 genes (Table S3). We then extracted the expression data of the 78 genes in 14 tissues (Fig. 5) and four different year-old roots (Fig. 6). The expressions of the genes varied by multiple fold among different tissues and different year-old roots. Although each enzyme is encoded by multiple genes, only a few of the genes, particularly 1–3, in a gene family was actively expressed in one tissue or at one stage of root growth and development. It was also observed that some of the genes, particularly 1–3, in a gene family expressed significantly higher than others. Different gene members of a gene family expressed differentially in different tissues or at different stages of root growth and development.

**Figure 7:**
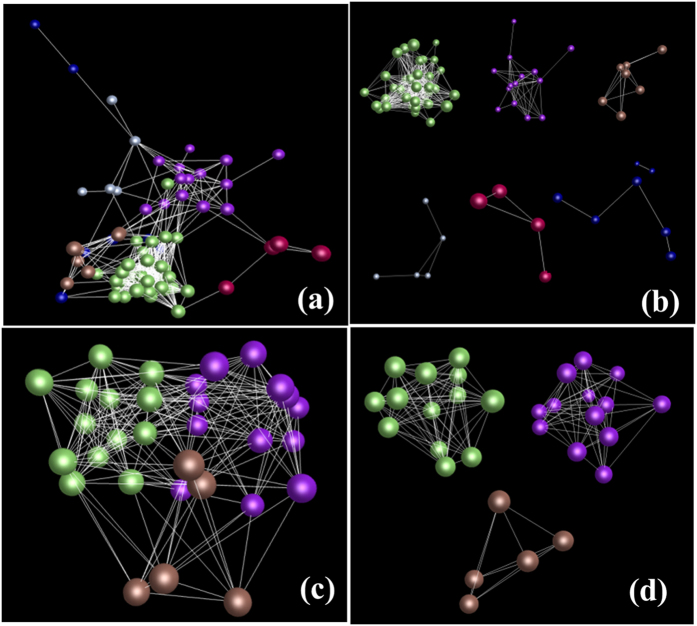
Network of genes likely involved in ginsenoside biosynthesis. (**a**) Co-expression network constructed from 78 genes likely involved in ginsenoside biosynthesis in different tissues at a cutoff of *P* ≤ 0.05, nodes = 74, edges = 565, clusters = 6. Different colors indicate different clusters that have closer interactions (also see Fig. 7b). (**b**) Different interaction clusters of the genes of the network. (**c**) Co-expression network constructed from 31 genes likely involved in ginsenoside biosynthesis in different year-old roots at a cutoff of *P* ≤ 0.05, nodes = 31, edges = 227, clusters = 3. Different colors indicate different clusters that have closer interactions (also see Fig. 7d). (**d**) Different interaction clusters of the genes of the network. For detail of the clusters, see Table S5.

**Figure 8:**
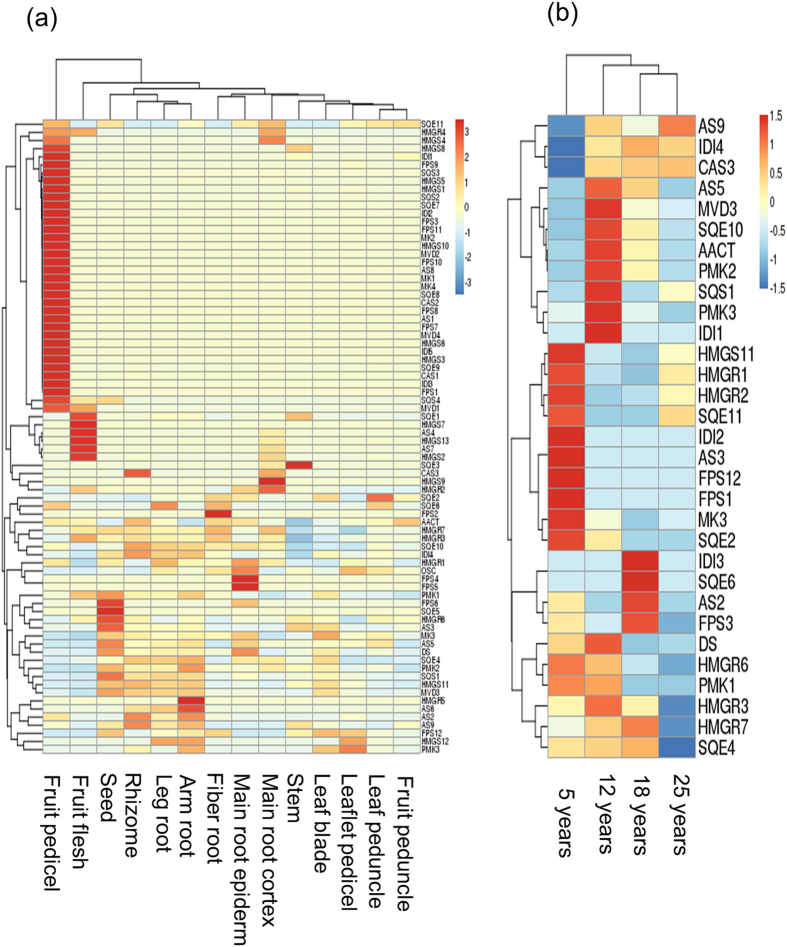
Heatmaps of genes likely involved in ginsenoside biosynthesis constructed based on their expressions in (a) 14 tissues and (b) different year-old roots.

We also characterized the expression relationships of the 78 genes by network analysis and the heatmap method. Of the 78 genes, 74 formed a single co-expression network in different tissues while 31 formed a single co-expression network at different stages of root growth and development (Fig. 7). These results suggest that the genes likely involved in the process of ginsenoside biosynthesis tended to be inter-correlated in expression. The heatmap analysis showed that different tissues or different year-old roots clearly had different expression profiles for the 78 genes (Fig. 8). The genes having similar expression levels were usually not from a same gene family, but from different gene families. This observation was especially apparent in different year-old roots (Fig. 8b).

## Discussion

The genes contained in a genome are basic for all biological aspects of an organism. We, for the first time, have sequenced the genes expressed in whole Jilin ginseng plant, profiled their expressions in different tissues and at different stages of root growth and development, and comprehensively analyzed the dynamics of the ginseng transcriptome in a view of systems biology. Analysis of the gene sequences and expressions has revealed several findings of interest. First, while the numbers of genes expressed in different tissues and different year-old roots vary dramatically (Table 1), the percentages of genes categorized into each GO term functional category are largely consistent among different tissues or different year-old roots, and are similar to those of whole plant (Fig. 1). These results indicate that it is essential for maintaining the normal activities of a cell to properly allocate the genes of an organism to different biological processes and to keep it consistent cross tissues and developmental stages. Second, nearly 90% of the genes in ginseng are one or more tissue-specific, while only approximately 40% are developmental stage-specific (Fig. 3). If the genes expressed in all tissues or all developmental stages of roots are considered to be house-keeping genes, nearly 60% of the genes are house-keeping, expressing consistently in roots through 20 years of their growth and development, the percentage being six fold of that in different tissues sampled at a single growth and development stage. These results suggest that the activities of the genes are much more consistent through different growth and development stages than in different tissues. Third, the genes contained in a ginseng cell are correlated in action, forming a co-expression network, even though the genes that are more related in functionality may be further grouped into closer interaction clusters (Fig. 4). In this study, a sample of only 5,000 unigenes randomly selected from the 248,993 unigene set was analyzed due to absence of a proper computing tool, but it could be predicted from this study that the co-expression network of the genes will be further enhanced, if more or all of the genes are analyzed for network construction. If the finding is true, it implies that all traits or biological processes, regardless of whether they are quantitative or qualitative in heritance, are controlled by multiple genes or consequences of multiple gene interactions. Fourth, the genes involved in same or functionally related pathways are correlated in action (Fig. 7). This may provide information useful for isolation of genes contributing to a same trait or biological process using a cloned gene controlling the trait. Finally, different genes of a gene family in ginseng express differentially in different tissues or at different developmental stages (Fig. 5). This indicates that different members of a gene family may be responsible for the functions of the gene family in different tissues or at different developmental stages. Since most of genes contained in an organism, especially in higher organisms, exist in multiple copies or a form of multiple gene families^34^, this finding may provide an insight into the importance and functionality of gene families in plants.

Genomic resources and tools, such as genes and gene expression profiles, are essential for many aspects of advanced research of an organism. Nevertheless, only 17,114 ESTs for Asian ginseng (*P. ginseng*) and 5,018 ESTs for American ginseng (*P. quinquefolius*) are currently available in GenBank (as of April 2015). Recently, a comprehensive unigene set of root transcriptome has been developed and a gene expression database was established for Asian ginseng^26^,^27^. This study provides not only a comprehensive set of 248,993 unigenes for Asian ginseng whole plant, in addition to the unigene sets of 14 different tissues and four different year-old roots (5-, 12-, 18- and 25-year-old), but also the expression profiles of every gene and isoform of the 248,993 unigene set in the 14 tissues and four different year-old roots. These results will significantly enhance the gene database of ginseng currently available in GenBank and the public^26^,^27^, and provide a comprehensive gene expression database. Moreover, since the sequences of the unigene sets were generated from 14 different tissues and four different year-old roots using a shotgun type approach, the unigene sets are expected to have a long coverage of ginseng’s functional genes and most, if not all, of the genes have full length^27^. Because the gene expression data generated in this study includes the expression profiles of every gene and isoform of the 248,993 unigene set in 14 different tissues sampled at the fruit ripening stage and in four different year-old roots sampled through 20 years, it is also comprehensive. Therefore, the gene sequences and expression profiles developed in this study also provide comprehensive resources and tools essential for advanced research and breeding of ginseng and related species.

Finally, the utility of the gene sequences and expression profiles developed in this study has been demonstrated by isolation and characterization of the genes that are potentially involved in ginsenoside biosynthesis. A total of 155 isoforms derived from 78 genes were isolated from the unigenes of the gene sequence data generated in this study that are likely to be involved in ginsenoside biosynthesis and classified into 13 different gene families. Furthermore, these genes have been characterized in expression and expression correlation in different tissues and at different stages of ginseng root growth and development. Although further studies, such as transformation analysis of the genes, will be needed to confirm the functions of the genes, the genes identified from the gene sequences provide candidates for isolation of the genes involved in ginsenoside biosynthesis. Analysis of the genes using the expression data has also led to findings of the functional diversification and action pattern of different members of a gene family.

## Conclusion

Medicinal plants are paramount to human health and medicine. Ginseng is the number one of medicinal herbs in North America and Asia. This study, for the first time, has comprehensively sequenced the expressed genes, profiled their expressions in 14 different tissues and four different stages of root development through 20 years, and characterized the ginseng transcriptome in a view of systems biology. Analysis of the genes and their expressions has revealed several findings of interest and provided an overall insight into the functions, dynamics and biology of the ginseng transcriptome. The sequences and expression profiles of the genes provide an encyclopedia for enhanced genetics and genomics research of ginseng and related medicinal plant species.

## Methods

### Plant materials and preparation

Four-year-old plants of Jilin ginseng cv. Damaya growing in a production field were sampled at the fruit ripening stage (late July 2010). The plants were divided into 14 parts (named as tissues in this article) as shown in Figure S1, immediately frozen in liquid nitrogen and stored at –80 °C. Similarly, root systems were sampled from 5-, 12-, 18- and 25-year-old plants of Jilin ginseng cv. Damaya planted in forest on the Changbai mountains, immediately frozen in liquid nitrogen and stored at –80 °C. The ages of the plants were determined according to the time that they were planted and further confirmed based on their morphology.

In some gene expression studies, biological replicates followed by qRT-PCR confirmation were deployed. However, our (in preparation) and other^35^ studies showed that digital gene expression profiling by high-throughput RNA sequencing is highly reproducible, with a correlation coefficient ranging from 0.90–0.98 (*P* = 0.000), no matter whether the samples were collected from different individuals growing in a replicate, in different replicates or in different years of an experiment, or no matter whether the experiment was carried out in environment-controlled or naturally field condition. Therefore, it is apparent that it is unnecessary to have biological replicates for digital gene expression profiling by high-throughput RNA sequencing. This conclusion is also supported by another digital gene expression profiling method (SAGE) facilitated by RNA sequencing using a traditional Sanger sequencer in which biological replicates were not usually applied^29^,^30^,^31^. Moreover, the validation of gene expression by qRT-PCR has also been a concern. First, RNA alternative splicing is a universal phenomenon, which is leading to multiple transcripts (isoforms) per gene that are different in sequence domains. Analysis showed that there were 1–136 isoforms per gene, with an average of approximately 2 isoforms per gene (Figure S2). Furthermore, our (not shown) and other^36^ studies showed that alternative splicing is tissue-specific, and the numbers and types of the isoforms for genes varied dramatically among tissues and across developmental stages. Therefore, while the expression of each isoform of a gene can be accurately measured by RNA sequencing, it cannot be done by either qRT-PCR or microarray because it is difficult to design qRT-PCR primers or microarray oligo probes to quantify expression of an isoform of a gene expressed in different tissues. This implies that the expression profiling results of both qRT-PCR and microarray may vary significantly, depending on where the qRT-PCR primers or microarray probes are designed in the target gene. Therefore, it is unreasonable to validate the gene expression results from RNA sequencing by qRT-PCR. Furthermore, because qRT-PCR is very low in throughput for gene expression profiling, only a few to dozens of genes could be analyzed to validate the gene expression results of high-throughput RNA sequencing. This number is less than 0.1% of the unigenes (e.g., 248,993) of a sample generated with high-throughput RNA sequencing, whose sampling size statistically is too small to meaningfully validate the gene expression results. Finally and importantly, as indicated above, the digital gene expression profiling by RNA sequencing itself is highly reproducible. Therefore, the samples collected from one biological replicate were analyzed and no qRT-PCR validation was performed for the digital expression profiling of the resulted isoforms so that the resources were used to sequence and analyze the transcriptomes of more tissues and more developmental stages in this study.

### RNA extraction

One hundred milligram of frozen samples of each tissue was ground into a fine powder in liquid nitrogen and total RNA was isolated using the Spectrum Plant Total RNA Kit (Sigma-Aldrich, St. Louis). The RNA was dissolved in 50 μl RNase-free TE (10 mM Tris, 1 mM EDTA, pH 8.0) and stored at −80 °C.

### RNA-Seq library construction and sequencing

RNA quality index (RQI) was determined using the Experion Automated Electrophoresis System (Bio-Rad, Hercules); only those having RQI ≥ 8.0 out of 10.0 for perfect integrity were used for RNA-seq library construction. RNA-Seq library for each sample was prepared using the TruSeq^TM^ RNA Sample Prep Kit v2 (illumina, San Diego), qualified and quantified using the Experion Automated Electrophoresis System (Bio-Rad, Hercules) and then, shipped on dry ice to Beijing Genomics Institute (BGI, Hong Kong) for sequencing. The libraries were subjected to quality check, pooled according to their nucleotide indexes and sequenced with Illumina HiSeq^TM^ 2000 using a 100PE module at BGI (Hong Kong). The clean reads were extracted with the Illumina pipelines, adaptor-trimmed with the BGI pipelines using the *SOAPnuke1.5.0* software that removes not only the adapters, but also low-quality reads (unpublished) and sorted according to indexes at BGI. The clean and trimmed reads of each sample were obtained from BGI.

### Unigene assembly and annotation

The Trinity software^37^,^38^ was used to assemble the unigene sets from the clean reads of all 14 tissues for whole plant, individual tissues and different year-old roots, separately, using the default parameters. We assembled the unigene sets for individual tissues and different year-old roots to estimate the variation of GO term profiles across tissues and developmental stages, which is significant for systematic understanding of ginseng’s transcriptome biology. The isoforms of rRNA genes contaminated in the unigene set was identified by blasting against the rRNA gene databases of all plant species available in GenBank. The isoforms derived from lncRNA were identified by ORF analysis because lncRNA has no or little ORF.

The unigenes were annotated using Blast2GO^39^ (https://www.blast2go.com/), a software package that retrieves GO terms, allowing gene functions to be determined and compared. The annotation was performed against the NCBI Nr database at a cutoff of E-value ≤ 1e-05. The GO terms were assigned to query sequences, producing a broad overview of groups of genes catalogued in the transcriptome for each of three ontological vocabularies: biological process, molecular function and cellular component. The three ontological vocabularies were then further categorized into different functional subcategories (level 2), such as metabolic process, response to stimulus, reproduction, signaling, etc., under the category of biological process.

The unigenes assigned to GO terms above were also mapped to KEGG pathways using the KEGG Database (http://www.genome.jp/kegg/) with Blast2GO. Enzyme commission (EC) numbers were assigned to the unigenes that had a BLASTX score of E-value ≤ 1e-05. The unigenes were mapped to the KEGG metabolic pathways according to the EC distribution in the pathway database.

### Gene expression profiling

The expressions of every isoform in different tissues and different year-old roots were profiled by the Trinity software^37^,^38^ with the RSEM (RNA-seq by Expectation Maximization) module using the 248,993 unigene set of whole plant as a reference. Because the same reference unigene set was used to profile the gene expression in all tissues and roots at different developmental stages, which excluded the influence of unigene length on gene expression profiling with the RNA sequencing method, the number of transcripts per million transcripts (TPM) was used to present the unigenes’ expression levels. Therefore, the levels of expression for a unigene could be compared directly among tissues or different stages of root growth and development, without need of additional normalization and additional same transcript search among tissues and developmental stages of roots.

### Construction of heatmaps and gene co-expression networks

The R programming language and software (http://www.r-project.org/) were used to calculate Spearman’s correlation coefficients for construction of heatmaps and gene co-expression networks. The heatmaps were constructed using the R programming language and software and the gene co-expression networks were constructed using the BioLayout Express^3D^ software^40^ (http://www.biolayout.org/).

## Additional Information

**Accession codes:** The clean reads data from 14 tissues and 4 different year-old roots of this study are submitted to the NCBI Gene Expression Omnibus (GEO) under accession number SRP066368 and the sequences of the unigenes are deposited at NCBI under BioProject PRJNA302556.

**How to cite this article**: Wang, K. *et al.* The Spatial and Temporal Transcriptomic Landscapes of Ginseng, *Panax ginseng* C. A. Meyer. *Sci. Rep.* **5**, 18283; doi: 10.1038/srep18283 (2015).

## Supplementary Material

Supplementary Datset

Supplementary Information

## Figures and Tables

**Table 1:** Statistics of unigene assemblies of whole plant, 14 tissues and four different year-old roots.

Tissues	No. of clean read (million)	No. of unigenes	Total length (nt)	N50 (nt)	Mean length (nt)
Whole plant (14 tissues)	199.0	248,992	230,567,518	1,572	936
Fiber root	10.7	60,912	46,100,736	1,156	756
Leg root	16.1	71,895	57,862,694	1,230	804
Main root epiderm	15.5	72,732	57,924,492	1,215	796
Main root cortex	16.8	76,214	61,479,490	1,240	806
Rhizome	13.2	69,706	56,673,470	1,249	813
Arm root	13.8	59,092	45,041,682	1,168	762
Stem	13.3	74,660	57,036,569	1,143	763
Leaf peduncle	15.3	80,046	68,206,781	1,331	851
Leaflet pedicel	12.4	68,607	52,869,647	1,171	770
Leaf blade	13.3	79,014	67,390,077	1,372	852
Fruit peduncle	13.7	78,205	64,946,485	1,288	830
Fruit pedicel	15.8	113,456	82,537,973	1,145	727
Fruit flesh	15.4	67,621	57,423,058	1,309	849
Seed	15.3	61,249	43,902,261	1,060	716
5-year-old root	16.3	63,926	52,654,535	1,246	823
12-year-old root	13.8	54,444	41,010,388	1,157	668
18-year-old root	15.5	56,696	45,997,394	1,239	717
25-year-old root	18.6	65,412	57,719,792	1,352	771

**Table 2:** Statistics of GO analysis and pathway mapping of the unigene sets derived from whole plant, different tissues and different year-old roots.

Tissue	No. of unigenes assigned to GO terms	No. of GO terms	No. of unigenes having enzyme codes	No. of enzyme codes	KEGG pathways
Whole plant (14 tissues)	109,781	716,067	29,321	35,819	138
Fiber root	32,916	223,563	8,851	10,784	135
Leg root	38,894	265,782	10,209	12,365	135
Main root epiderm	38,141	261,895	10,295	12,423	137
Main root cortex	40,396	283,052	11,422	13,746	137
Rhizome	37,901	259,518	10,232	12,253	135
Arm root	32,179	219,031	8,381	10,191	136
Stem	38,260	259,573	10,281	12,417	136
Leaf peduncle	41,162	285,345	10,961	13,457	137
Leaflet pedicel	36,327	248,875	9,624	11,492	136
Leaf blade	39,980	271,898	10,901	13,210	135
Fruit peduncle	40,229	279,176	10,768	13,030	137
Fruit pedicel	58,557	385,996	16,106	19,502	138
Fruit flesh	36,354	246,855	9,304	11,313	134
Seed	31,311	182,789	8,079	9,417	133
5-year-old root	37,317	252,188	9,657	11,701	136
12-year-old root	32,306	217,553	8,409	10,238	135
18-year-old root	32,787	219,384	8,417	10,274	135
25-year-old root	38,202	254,454	9,723	11,773	137
