# Supplementary material for: The Spatial and Temporal Transcriptomic Landscapes of Ginseng, Panax ginseng C. A. Meyer
Source: Sci Rep. 2015 Dec 11;5:18283. doi: 10.1038/srep18283 (PMC4675998; doi:10.1038/srep18283)
Supplement: Supplementary Information [file srep18283-s2.pdf]

# Supplementary information

## The Spatial and Temporal Transcriptomic Landscapes of Ginseng, *Panax ginseng* C. A. Meyer

**Kangyu Wang, Shicui Jiang, Chunyu Sun, Yanping Lin, Rui Yin, Yi Wang* and Meiping Zhang***

College of Life Science, Jilin Agricultural University, Changchun 130118, Jilin, China

**Table S1.** Expressions of the 248,993 unigenes in different tissues and different year-old roots of Jilin ginseng. The gene expression level is presented in number of transcripts per million (TPM) (see Supplementary Dataset)

**Table S2.** The numbers of genes specifically expressed in one or two of the 14 tissues studied

| Tissue | A | B | C | D | E | F | G | H | I | J | K | L | M | N |
|---|---|---|---|---|---|---|---|---|---|---|---|---|---|---|
| A. Fiber root | **892** | 607 | 179 | 184 | 169 | 308 | 146 | 155 | 127 | 208 | 151 | 586 | 151 | 120 |
| B. Leg root | | **1,258** | 527 | 202 | 255 | 402 | 160 | 193 | 157 | 278 | 214 | 276 | 141 | 156 |
| C. Main root epiderm | | | **1,430** | 354 | 283 | 163 | 264 | 259 | 187 | 303 | 246 | 337 | 146 | 183 |
| D. Main root cortex | | | | **1,687** | 302 | 122 | 376 | 299 | 216 | 492 | 346 | 1,286 | 227 | 270 |
| E. Rhizome | | | | | **1,162** | 170 | 252 | 234 | 179 | 302 | 205 | 847 | 171 | 171 |
| F. Arm root | | | | | | **890** | 148 | 144 | 121 | 140 | 128 | 1,041 | 124 | 123 |
| G. Stem | | | | | | | **1,445** | 392 | 218 | 429 | 319 | 1,197 | 219 | 238 |
| H. Leaf peduncle | | | | | | | | **1,524** | 340 | 673 | 454 | 1,326 | 265 | 205 |
| I. Leaflet pedicel | | | | | | | | | **1,198** | 448 | 293 | 326 | 179 | 163 |
| J. Leaf blade | | | | | | | | | | **2,349** | 714 | 526 | 361 | 238 |
| K. Fruit peduncle | | | | | | | | | | | **1,483** | 491 | 218 | 227 |
| L. Fruit pedicel | | | | | | | | | | | | **33,169** | 2,664 | 1,039 |
| M. Fruit flesh | | | | | | | | | | | | | **1,246** | 598 |
| N. Seed | | | | | | | | | | | | | | **1,436** |

**Table S3.** List of the candidate genes likely involved in ginsenoside biosynthesis

| Gene | Name | Enzyme code | No of genes |
|---|---|---|---|
| *AACT* | Acetyl-CoA acetyltransferase | 2.3.1.9 | 1 |
| *HMGS* | HMG-CoA synthase | 2.3.3.10 | 13 |
| *HMGR* | HMG-CoA reductase (NADPH) | 1.1.1.34 | 7 |
| *MK* | Mevalonate kinase | 2.7.1.36 | 4 |
| *PMK* | Phosphomevalonate kinase | 2.7.4.2 | 3 |
| *MVD* | Mevalonate diphosphate decarboxylase | 4.1.1.33 | 4 |
| *IDI* | Isopentenyl-diphosphate-isomerase | 5.3.3.2 | 5 |
| *FPS* | Farnesyl diphosphate synthase | 2.5.1.1/10 | 12 |
| *SQS* | Squalene synthase | 2.5.1.21 | 4 |
| *SQE* | Squalene epoxidase | 1.14.13.132 | 11 |
| *DS* | Dammarenediol-II synthase | 4.2.1.125 | 1 |
| *AS* | β-amyrin synthase | 5.4.99 | 9 |
| *OSC, CAS* | Oxidosqualene cyclase/cycloartenol synthase | 5.4.99.7/8 | 1, 3 |

**Table S4.** The genes likely involved in ginsenoside biosynthesis and expressed in different tissues and different year-old roots

| Tissue | Number | Gene |
|---|---|---|
| Fiber root | 3 | HMGS12,HMGR1,SQE6 |
| Leg root | 7 | HMGS12,PMK3,SQE4,PMK2,HMGR5,AS6,AS2 |
| Main root epiderm | 2 | FPS12,MK3 |
| Main root cortex | 3 | SQE1,SQE3,AS9 |
| Rhizome | 11 | SQS1,AS5,DS,MK3,HMGS11,MVD3,PMK1,FPS6,SQE5,HMGR6,AS3 |
| Arm root | 7 | AS9,CAS3,AS2,SQE10,IDI4,HMGS11,AACT |
| Stem | 3 | HMGS12,PMK3,OSC |
| Leaf peduncle | 1 | SQE2 |
| Leaflet pedicel | 3 | SQE6,HMGR7,FPS2 |
| Leaf blade | 5 | DS,HMGR1,OSC,FPS4,FPS5 |
| Fruit peduncle | 2 | AACT,HMGR3 |
| Fruit pedicel | 34 | SQE11,HMGS8,IDI1,FPS9,SQS3,HMGS5,HMGS1,SQS2,HMGS3,SQE9,IDI2,FPS3,FPS11,MK2,HMGS10,MVD2,FPS10,AS8,MK1,MVD4,HMGS6,IDI5,CAS1,MK4,SQE7,SQE8,CAS2,FPS8,AS1,FPS7,IDI3,FPS1,SQS4,MVD1 |
| Fruit flesh | 6 | CAS3,SQE11,HMGR4,HMGS4,HMG9,HMGR2 |
| Seed | 9 | MVD1,HMGR3,HMGS7,AS4,HMGS13,AS7,HMGS2,HMGR4,HMGR2 |
| 5 year-old root | 13 | DS,SQE11,HMGS11,HMGR1,IDI2,AS3,FPS12,FPS1,MK3,SQE2,HMGR2,HMGR6,PMK1 |
| 12 year-old root | 8 | IDI4,HMGR7,AS2,FPS3,PMK2,CAS3,IDI3,SQE6 |
| 18 year-old root | 1 | SQE1 |
| 25 year-old root | 8 | HMGR3,PMK3,IDI1,SQS1,AS5,MVD3,AACT,SQE10 |

**Table S5.** List of genes contained in each cluster of the gene co-expression networks shown in Figure 7

| Cluster | Number | Gene |
|---|---|---|
| | | **Different tissues** |
| Cluster01 | 37 | SQS4,SQS3,SQS2,SQE9,SQE8,SQE7,SQE11,OSC,MK4,MK2,MK1,MVD4,MVD2,MVD1,IDI5,IDI3,IDI2,IDI1,HMGS8,HMGS6,HMGS5,HMGS4,HMGS3,HMGS10,HMGS1,HMGR4,FPS9,FPS8,FPS7,FPS3,FPS11,FPS10,FPS1,CAS2,CAS1,AS8,AS1 |
| Cluster02 | 14 | SQS1,SQE4,SQE3,PMK2,MK3,MVD3,IDI4,HMGS11,FPS12,DS,AS9,AS5,AS3,AACT |
| Cluster03 | 7 | HMGS9,HMGS7,HMGS2,HMGS13CAS3,AS7,AS4 |
| Cluster04 | 5 | SQE10,HMGR5,HMGR1,AS6,AS2 |
| Cluster05 | 4 | SQE5,FPS6,FPS5,FPS4 |
| No class | 7 | SQE1,PMK3,HMGS12,HMGR7,HMGR6,HMGR3,HMFR2 |
| | | **Different year-old roots** |
| Cluster01 | 13 | SQE4,SQE11,SQE10,PMK2,MVD3,IDI1,HMGS11,HMGR7,HMGR3,HMGR2,HMGR1,AS5,AACT |
| Cluster02 | 13 | SQE2,PMK3,MPK1,MK3,IDI4,IDI2,HMGR6,FPS12,FPS1,DS,CAS3,AS9,AS3 |
| Cluster03 | 5 | SQS1,SQE6,IDI3,FPS3,AS2 |

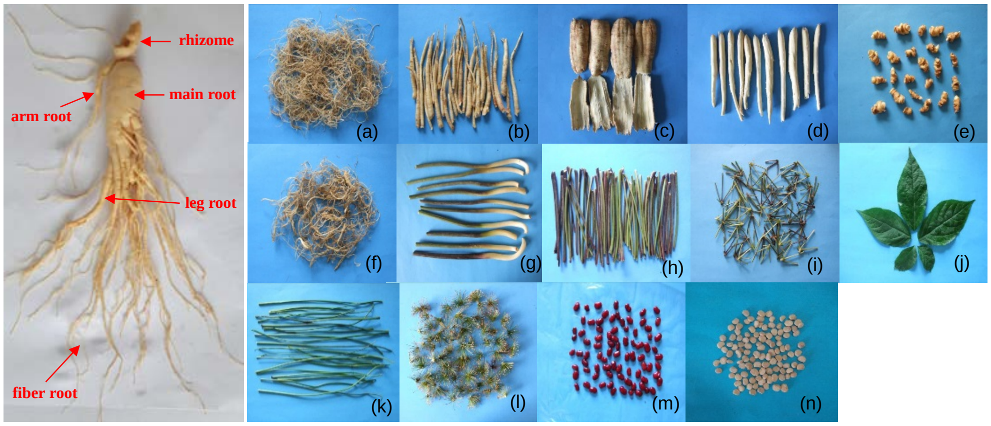


**Figure S1.** Fourteen tissues of Jilin Ginseng plants used in this study. (a) fiber root; (b) leg root; (c) main root epiderm; (d) main root cortex; **(**e) rhizome; **(**f) arm root; (g) stem; (h) leaf peduncle; (i) leaflet pedicel; (j) leaf blade; (k) fruit peduncle; (l) fruit pedicel; (m) fruit flesh and (n) seed.

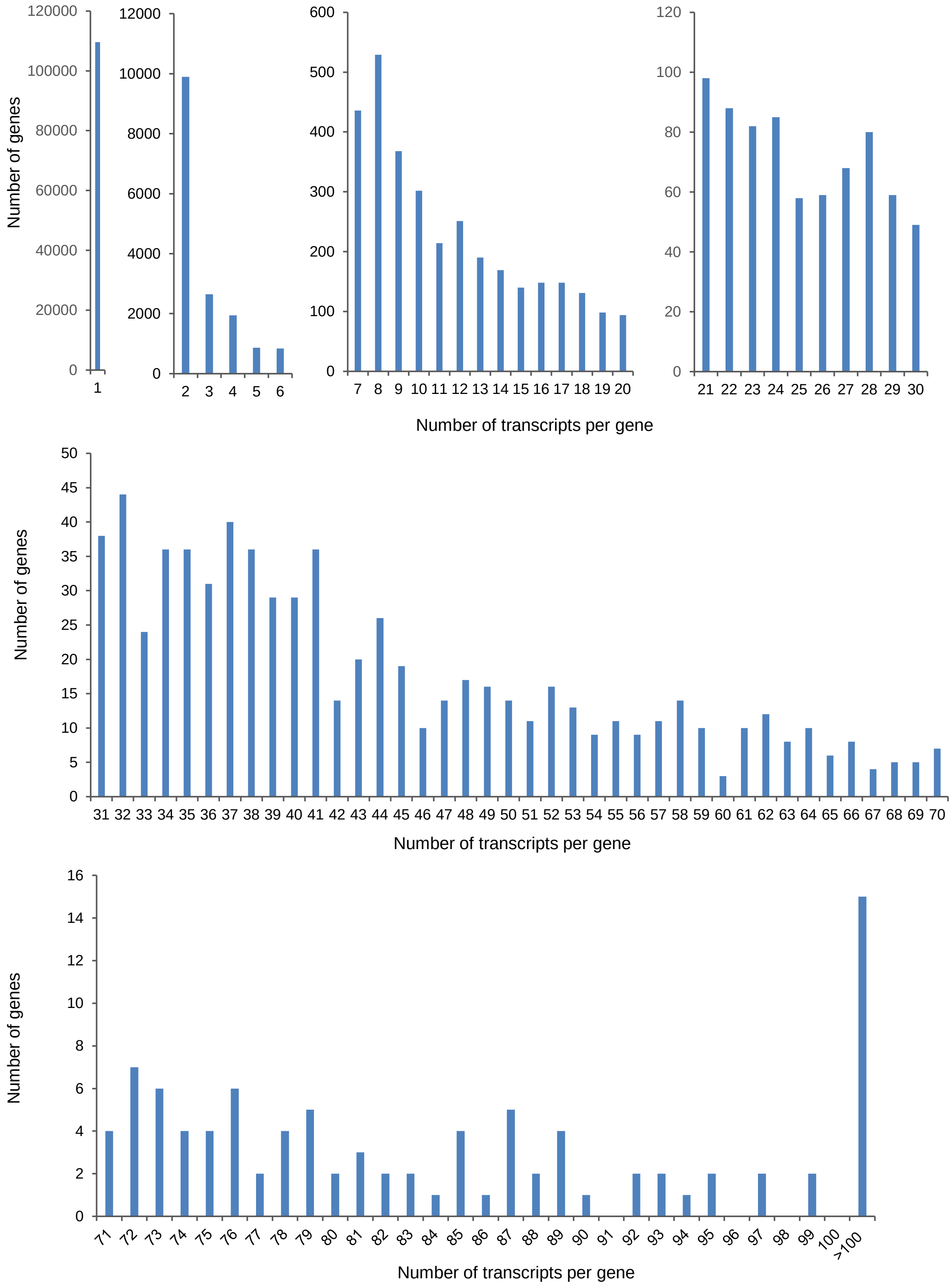


**Figure S2.** Distribution of gene models having different numbers of transcripts per gene resulting from alternative splicing.
